# Key drivers for market penetration of biosimilars in Europe

**DOI:** 10.1080/20016689.2016.1272308

**Published:** 2017-01-30

**Authors:** Cécile Rémuzat, Julie Dorey, Olivier Cristeau, Dan Ionescu, Guerric Radière, Mondher Toumi

**Affiliations:** ^a^ Pricing and Market Access Department, Creativ-Ceutical, Paris, France; ^b^ HEOR Department, Creativ-Ceutical, Paris, France; ^c^ Global Pricing and Market Access Biopharmaceuticals Department, Sandoz International GmbH, Holzkirchen, Germany; ^d^ Faculté de Médecine, Laboratoire de Santé Publique, Aix-Marseille Université, Université de la Méditerranée, Marseille, France

**Keywords:** Biosimilar, uptake, Europe, price erosion, market dynamics, incentive policies

## Abstract

**Background** & **Objectives**: Potential drivers and barriers of biosimilar uptake were mainly analysed through qualitative approaches. The study objective was to conduct a quantitative analysis and identify drivers of biosimilar uptake of all available biosimilars in the European Union (EU).

**Methods**: A three-step process was established to identify key drivers for the uptake of biosimilars in the top 10 EU member states (MS) pharmaceutical markets (Belgium, France, Germany, Greece, Hungary, Italy, Poland, Spain, Sweden, and the UK): (1) literature review to identify incentive policies in place to enhance biosimilars adoption; (2) assessment of biosimilar market dynamics based on database analysis; (3) regression model analysis on price using the following explicative variables: incentive policies; price difference between the biosimilar and the originator product; distribution channel; generic uptake and generic price cut; pharmaceutical expenditure per capita; and market competition.

**Results**: At the study cut-off date, 20 biosimilars were available on the market. Incentive policies applied to biosimilars were found to be heterogeneous across countries, and uptakes of biosimilars were also very heterogeneous between different therapeutic classes and countries. Results from the model demonstrated that incentive policies and the date of first biosimilar market entry were correlated to biosimilar uptake. Pharmaceutical expenditure per capita and the highest generic uptake were inversely correlated with biosimilar uptake. Average generic price discount over originator and the number of biosimilars showed a trend toward statistical significance for correlation with biosimilar uptake, but did not reach the significance threshold. Biosimilar price discount over original biologic price, the number of analogues, and the distribution channel were not correlated with the biosimilar uptake.

**Conclusions**: Understanding drivers of biosimilar uptake becomes a critical issue to inform policy decision-makers. This study showed that incentive policies to enhance uptake remain an important driver of biosimilar penetration, while biosimilar price discounts have no impact. Future research is warranted when the biosimilar market gains maturity.

## Background and objective

The uptake of biosimilar medicines is a key and highly debated topic in the European Union (EU). With the first biosimilar approved in 2006 [1], the EU is the global pioneer in the approval of biosimilars and the most developed market in this area. As of March 2016, 20 biosimilars were available on the market (Table 1) representing six therapeutic classes (i.e., granulocyte-colony stimulating factor [G-CSF], epoetin [EPO], insulin, anti-tumour necrosis factor [Anti-TNF], gonadotropins, and human growth hormone [HGH] [1]).

**Table 1.** List of biosimilars approved by the European Medicines Agency (as of March 2016) [1].

Product class (number of biosimilars)	Reference product brand name (Company)	Biosimilar brand name	International non-proprietary name	Marketing authorisation holder (MAH)	Marketing authorisation date
Granulocyte-colony stimulating factor (G-CSF) (8 biosimilars)	Neupogen® (Amgen)	Accofil®	filgrastim	Accord Healthcare Ltd	18 September 2014
	Neupogen® (Amgen)	Grastofil®	filgrastim	Apotex Europe BV	18 October 2013
	Neupogen® (Amgen)	Nivestim®	filgrastim	Hospira UK Ltd	8 June 2010
	Neupogen® (Amgen)	Zarzio®	filgrastim	Sandoz GmbH	6 February 2009
	Neupogen® (Amgen)	Filgrastim Hexal®	filgrastim	Hexal AG	6 February 2009
	Neupogen® (Amgen)	Biograstim®	filgrastim	AbZ-Pharma GmbH	15 September 2008
	Neupogen® (Amgen)	Ratiograstim®	filgrastim	Ratiopharm GmbH	15 September 2008
	Neupogen® (Amgen)	Tevagrastim®	filgrastim	Teva GmbH	15 September 2008
	*Neupogen® (Amgen)*	*Filgrastim ratiopharm®*	*filgrastim*	*Ratiopharm GmbH*	*15 September 2008* *(withdrawn from market at request of MAH)*
Epoetin (5 biosimilars)	Eprex/Erypo® (Janssen)	Retacrit®	epoetin zeta	Hospira UK Limited	18 December 2007
	Eprex/Erypo® (Janssen)	Silapo®	epoetin zeta	Stada Arzneimittel AG	18 December 2007
	Eprex/Erypo® (Janssen)	Abseamed®	epoetin alfa	Medice Arzneimittel Pütter GmbH & Co. KG	28 August 2007
	Eprex/Erypo® (Janssen)	Epoetin alfa Hexal®	epoetin alfa	Hexal AG	28 August 2007
	Eprex/Erypo® (Janssen)	Binocrit®	epoetin alfa	Sandoz GmbH	28 August 2007
Insulin (1 biosimilar)	Lantus® (Sanofi)	Abasaglar®	insulin glargine	Eli Lilly Regional Operations GmbH	9 September 2014
Anti-Tumour Necrosis Factor (Anti-TNF) (3 biosimilars)	*Enbrel® (Pfizer)^a^*	*Benepali®*	*etanercept*	*Samsung Bioepis UK Limited (SBUK)*	*14 January 2016*
	Remicade® (Janssen)	Inflectra®	infliximab	Hospira UK Limited	10 September 2013
	Remicade® (Janssen)	Remsima®	infliximab	Celltrion Healthcare Hungary Kft.	10 September 2013
Gonadotropins (2 biosimilars)	Gonal-f® (Merck Serono)	Bemfola®	follitropin alfa	Finox Biotech AG	27 March 2014
	Gonal-f® (Merck Serono)	Ovaleap®	follitropin alfa	Teva Pharma B.V.	27 September 2013
Human Growth Hormone (HGH) (1 biosimilar)	*Humatrope® (Eli Lilly)*	*Valtropin®*	*somatropin*	*BioPartners GmbH*	*24 April 2006* *(withdrawn from market at request of MAH)*
	Genotropin® (Pfizer)	Omnitrope®	somatropin	Sandoz GmbH	12 April 2006

Note: ^a^Not included in the analysis.

The biosimilar market is expected to grow substantially, as best-selling biologics are scheduled to come off-patent in the coming years (Table 2) [2,3].

**Table 2.** **L**ist of best-selling biologics with patent expiry in the years to come [2,3].

International non-proprietary name	Brand name	Initial market authorisation date (EU/UK)	EU patent expiry date	Categories of licensed indications (as per summary of product characteristics)
Adalimumab	Humira®	8 September 2003	2018	Rheumatoid arthritis/juvenile idiopathic arthritis/enthesitis-related arthritis/psoriatic arthritis/axial spondyloarthritis/ankylosing spondylitis/psoriasis/Crohn’s disease/ulcerative colitis
Bevacizumab	Avastin®	12 January 2005	2019 [2]/2022 [3]	Carcinoma of the colon or rectum/breast cancer/Lung cancer/renal-cell cancer/Epithelial ovarian, fallopian tube, primary peritoneal cancer/carcinoma of the cervix
Cetuximab	Erbitux®	29 June 2004	2014	Colorectal cancer/cancer of the head and neck
Darbepoetin alfa	Aranesp®	8June 2001	2016	Anaemia
Enoxaparin	Lovenox®	January 1999	2012	Anticoagulant
Interferon beta-1	Avonex® Rebif®	13 March 1997 4 May 1998	2015	Multiple sclerosis Multiple sclerosis
Palivizumab	Synagis®	13 August 1999	2015	Lower-respiratory-tract disease
Pegfilgrastim	Neulasta®	22August 2002	2017	Neutropenia
Ranibizumab	Lucentis®	22 January 2007	2016	Age-related macular degeneration/visual impairment
Rituximab	MabThera®	2 June 1998	2013	Non-Hodgkin’s lymphoma/chronic lymphocytic leukaemia/rheumatoid arthritis/granulomatosis with polyangiitis and microscopic polyangiitis
Trastuzumab	Herceptin®	28 August 2000	2014	Breast cancer/Gastric cancer

In this perspective, the European Commission is actively working on the impact and promotion of biosimilar uptake within the EU through its Project Group on Market Access and Uptake of Biosimilars. The group involves EU member states (MS), European Economic Area (EEA) countries’ representatives, as well as other stakeholders such as patient organisations’ and health care professionals’ representatives, and experts [4,5].

As a consequence of the current cost-contained environment, the growing budget impact of costly biologic medicines – about 27% of pharmaceutical sales in Europe in 2014 [2] – is thoroughly scrutinised by healthcare payers. In an analogy with the introduction of generic drugs for small molecules, biosimilar medicines are expected to generate savings on the pharmaceutical expenditure of existing biologic medicines following patent expiry, thus sparing budget for innovative medicines. Additionally, biosimilars will enhance patient access to this category of medicines generally indicated for the treatment of severe diseases such as cancer, metabolic disorders, or immunology and inflammatory diseases [4,6]

The European Commission noticed in its recent *Workshop on Access to and Uptake of Biosimilars* that biosimilar uptake and competition varies across EU MS according to the therapeutic class of the biologic drugs and the different country policies. Based on these considerations, the European Commission is calling for better control of healthcare budgets and reduction of health inequalities between and within EU MS through an increasing competition between biologic medicines, including biosimilars [7].

Literature reported limited research on biosimilar competition, uptake, and impact on price erosion before 2010 [8], and more publications were released thereafter. Several studies have modelled the biosimilar competition based on the experience with generic competition and taking into account the differences between the two categories of products. The findings predicted lower price difference versus originator prices than with generics (Grabowski et al. [9]; Chauhan et al. [10]). The literature suggests a totally different market dynamic between generics and biosimilars in terms of higher development, production, and physicians’ education investments on biologic products, as well as different supply-side and demand-side policies across EU MS [11]. One study estimated the impact of biosimilar entry in terms of healthcare systems savings between 2007 and 2020 for eight EU countries (France, Germany, Italy, Poland, Romania, Spain, Sweden, and the UK), ranging from €11.8 billion to €33.4 billion [12]. Other qualitative studies assessed the levers and barriers to the uptake of biosimilars based on analysis of biosimilar uptake, price reduction, and various policies implemented across EU MS [2,8,13–17] One quantitative study (Rovira et al. [18]) assessed market dynamics of biosimilars in 24 EU MS, as well as Norway and Switzerland, between 2007 and 2010 using bivariate regression, and reported faster launch of biosimilars after marketing authorisation, associated to countries’ gross national income and expenditure on health, pharmacists’ generics substitution (inversely), and medicines’ price level index. A recent survey providing an overview of pricing and reimbursement policy approaches across Europe for biological medicines, and specifically applied to biosimilars showed heterogeneity in policies and their implementation between the different countries [11].

Several factors have been reported as potential drivers of biosimilar uptake and might explain the differences observed between EU MS, i.e., physician and patient adoption of biosimilars, national healthcare systems specificities in terms of pricing, reimbursement, and procurement policies [4,19] Bocquet et al. [16,20] reported that price difference between originator and biosimilar was not a main driver of EPO and G-CSF uptake; a low biosimilar uptake was partly explained by price cut of originators prior to biosimilar entry, as well as the development of new generation products competing with biosimilars. A recent study has shown a weak correlation between biosimilar uptake and price reduction [21]. Conversely, it was reported that ‘generic drug culture’ might be correlated with a better biosimilar adoption [22]. Biosimilar uptake also differs between therapeutic classes as a consequence of various drivers; an analysis showing the heterogeneity of biosimilar G-CSF uptake in EU-5 suggested the country differences in distribution channels as a potential factor [16]; however, this has not been shown for the EPO market [20]. These studies also suggested that incentives might impact differently the uptake of different product classes [16,20].

Although several studies described the potential drivers and barriers of biosimilar uptake, a qualitative approach was used in most of them, and assessed correlation between biosimilar market dynamics and various country characteristics such as pharmaceutical policies and economic features. Moreover, these studies were generally conducted when few biosimilars were on the market. In previous studies, authors did not attempt to identify the drivers of biosimilar uptake in a multivariate model integrating discount to biologic originator and incentives for adoption of biosimilars. While the biosimilar market environment is evolving fast, quantifying the potential drivers of biosimilar uptake might better inform stakeholders on the key levers to enhance access to more affordable biologics in EU.

In this context, the objective of this study was to conduct a quantitative analysis of the impact of biosimilar incentive policies on the uptake of all available biosimilars in the EU in light of other variables, including price difference between the biosimilar and the originator product, distribution channel, generic uptake and generic price cut, pharmaceutical expenditure per capita, and market competition.

## Methods

A three step process was established to identify the key drivers for the uptake of biosimilars: (1) a literature review to identify incentive policies in place to enhance biosimilars adoption in the selected countries; (2) assessment of biosimilar market dynamics based on database analysis; and (3) regression model analysis.

All biosimilars approved by the EMA between 2006 to 1 January 2016 were considered in this analysis (Table 1). Countries included were the 10 EU members of the Organisation for Economic Co-operation and Development (OECD) having the highest pharmaceutical expenditure (Table 3) [23,24], with the exception of the Netherlands, for which we did not have access to market data. Sweden was considered instead as an alternative North European country with substantial pharmaceutical spending. Selected countries were: Belgium; France; Germany; Greece; Hungary; Italy; Poland; Spain; the UK; and Sweden.

**Table 3.** Selection of the 10 EU countries members of the Organisation for Economic Co-operation and Development (OECD) having the highest pharmaceutical expenditure [23,24].

Country	Population (million persons), 2012	Pharma expenditure per capita, 2012 (at current prices and PPPs)	Pharma expenditure (million US$) = population*pharma expenditure per capita
Austria	8.4	561	4,712
Belgium	11.1	736	8,170
Czech Republic	10.5	439	4,610
Denmark	5.6	295	1,652
Estonia	1.3	311	404
Finland	5.4	473	2,554
France	63.5	651	41,339
Germany	80.4	668	53,707
Greece	11.1	599	6,649
Hungary	9.9	574	5,683
Ireland	4.6	666	3,064
Italy^c^	60.2	496	29,859
Luxembourg	0.5	399	200
*Netherlands*	*16.8*	*450*	*7,560*
Poland	38.5	321	12,359
Portugal^b^	10.6	473.3	5,017
Slovak Republic	5.4	535	2,889
Slovenia	2.1	513	1,077
Spain^b^	46.7	522.9	24,419
Sweden	9.5	478	4,541
United Kingdom^a^	60.5	367	2,2204

Notes: *2008; ^b^2011; ^c^2013. Italic values indicate countries not selected for the research, due to market data being inaccessible. Shaded rows indicate countries selected for the research.

### Literature review

A comprehensive literature review was conducted to identify publications describing incentive policies (policies towards physicians, pharmacists, and patients) in place for biosimilars in the 10 selected countries. The search strategy used free search terms and was performed between October and November 2015. The full description of the methodology for the literature review was presented elsewhere [25].

The search strategy was performed in the following databases: MEDLINE; Embase; The Cochrane library; Generics and Biosimilars initiative (GaBi) journal and website; and the International Society for Pharmacoeconomics and Outcomes Research (ISPOR) and Health Technology Assessment International (HTAi) websites for conference abstracts. This review was completed by additional searches in National Health Authorities and Parliament websites, Google, and Google Scholar, as well as grey literature. The search was conducted in the English language for all databases, except in country-specific databases (National Health Authorities and Parliament websites) which were searched using local languages. Publications were searched from 2005 (the year of establishment of a regulatory framework for the development of biosimilars by the European Medicines Agency [26]) to November 2015.

In a second stage, publications were screened and selected for relevance to the topic, and the full-text papers were reviewed in detail in order to identify incentive policies, and were classified as follows: (1) physicians’ incentives, i.e., pharmaceutical prescription budgets, prescription quotas, monitoring of prescriptions patterns, financial incentives or penalties, prescription conditions/guidelines, switching, INN prescribing, education/information; (2) pharmacists’ incentives, i.e., substitution right, financial incentives or penalties, education/information; and (3) patients’ incentives, i.e., patient co-payment, education/information.

### Database analysis

The objectives of this step were to identify the market size for each therapeutic class (all products of the class considered together) and assess the overall biosimilar market share and price discounts versus originator products.

Two commercial databases providing data for the 10 selected countries were used. Data on sales in value (US dollars) and in volume (standard unit defined by IMS as the number of units – tablets, capsules, ml or grams multiplied by the number of packages and package size and divided by smallest common dose of a product) for all considered products were extracted from the IMS MIDAS (Multinational Integrated Data Analysis System) database (with permission). Data were provided separately for hospital and retail pharmacy; with the exception of Greece, for which only retail sales were available, and Sweden, for which retail and hospital were combined. Data extraction covered the period from the first quarter of 2006 to the second quarter of 2015. Market launch dates as dates of first identified sale in IMS MIDAS were also collected. Data on official listed prices without discounts for all dosages, formulation, and pack sizes were extracted from the IMS Pricing Insights database (with permission). Manufacturer and retail listed prices were provided from launch to last update (extraction cut-off date: November 2015). Defined daily dose (DDD) for each product as defined by the WHO Collaborating Centre for Drug Statistics Methodology (http://www.whocc.no/) was extracted.

For each country and for each therapeutic class, uptakes of biosimilar products in value and in volume were calculated as the sales percentage of biosimilars over the sales percentage of originator products and biosimilars together. Sales of the last two quarters of 2014 and the first two quarters of 2015 were included in the calculation, irrespectively of dosage, formulation, and pack size. Uptakes in volume were expressed in standard unit, while uptakes in sales were in US dollars.

For each country and for each therapeutic class, biosimilar price discounts were calculated as the differences between the average price per DDD of biosimilars available on the market and the average price per DDD of originator products. This means that if more than one dosage and more than one pack size were available, the price per DDD was calculated for each one. Then, for each therapeutic class, average prices of all biosimilars and originator were computed using the last available ex-factory prices.

### Regression model development and analyses

In order to identify the drivers of biosimilar uptake, its relationship with several variables were analysed using regression models.

#### Covariates considered in the models

1. Biosimilar price discounts versus originator products by country and by therapeutic class.
2. Incentive policies (sum of number of incentives available within each country) which summed by country the total number of incentives: pharmaceutical prescription budgets; prescription quotas; monitoring of prescriptions patterns; financial incentives or penalties; prescription conditions/guidelines; switching; INN prescribing; education/information; substitution right; patient co-payment.
3. Market competition included in the model as the total number of biosimilar and analogue products by therapeutic class (described in Table 4).
4. Distribution channel by therapeutic class. Products were classified as hospital products if more than 75% of sales in volumes were made through hospital channel, as retail products if more than 75% of sales were made through retail channel, and as mixed retail–hospital products in other cases.
5. Six therapeutic classes were considered: G-CSF; EPO; insulin; Anti-TNF; gonadotropins; and HGH.
6. Generic uptake estimated in each country as the proportion of overall off-patent product sales based on IMS data from a IGES Institut report from 2015 on generics (small molecule) [27] (Table 5).
7. Average generic price discount at the time of generic entry based on data from literature and Creativ-Ceutical proprietary in-house database by country (Table 5).
8. Pharmaceutical expenditure per capita ($US) by country.
9. Market entry date of the first biosimilar of each therapeutic class.

**Table 4.** Analogue products considered in the study.

Product class (ATC4)	International non-proprietary name
Granulocyte-colony stimulating factor (G-CSF) (L03AA)	Lenograstim
	Lipegfilgrastim
	Molgramostim
	Pegfilgrastim
	Sargramostim
Epoetin (B03XA)	Epoetin beta
	Epoetin theta
	Epoetin delta
	Darbepoetin alfa
Insulin (A10AB-F)	Insulin glargine (Toujeo®; more concentrated version of Lantus®)
	Insulin aspart
	Insulin degludec
	Insulin detemir
	Insulin glulisine
	Insulin lispro
	Insulin human
Anti-tumour necrosis factor (Anti-TNF) (L04AB)	Adalimumab
	Certolizumab Pegol
	Etanercept
	Golimumab
Gonadotropins (G03GA)	Choriogonadotropin alfa
	Chorionic gonadotrophin
	Corifollitropin alfa
	Follitropin alfa/Lutropin alfa
	Follitropin beta
	Human menopausal gonadotrophin
	Lutropin alfa
	Urofollitropin
Human growth hormone (HGH) (H01AC)	Somatropin (other than Genotropin® and Humatrope®)

**Table 5.** Estimations of generic uptake versus off-patent market (volume) and generic discounts versus reference product used in the regression model.

Country	Generic uptake vs off-patent (from IGES report 2015 [26])	Generic price discount vs reference product (at first generic entry)
Belgium	41%	32.5% [28]
France	59%	60% [29]
Germany	81%	30%^a^ [30]
Greece	7%	35% [31]
Hungary	37%	40% [32]
Italy	41%	30–75% [33]
Poland	55%	25% [34]
Spain	58%	40% [35]
Sweden	64%	65% [36]
UnitedKingdom	71%	75% [37] Free pricing (price below off-patent reference product price) [65]

Note: ^a^Mandatory 10% rebate for generics unless the price is 30% below the reference price.

#### Statistical analyses

Several generalised linear models (GLM) were tested with different distributions (normal, beta, gamma, Poisson, binomial negative) and different link functions (identity and logarithm). The covariates incentive policies, distribution channel, therapeutic class were considered as categorical variables in the models. The variables country and therapeutic class were tested as random effects. In order to select among all tested models the model which best fit the data, two criteria were predefined to the analysis and used. The Akaike information criterion (AIC) was used to compare models when their structures were the same (same distribution, same link function); otherwise, the smallest root-mean-square error (RMSE) criterion was used. The model with the least criterion values was selected as the best model i.e., the model for which the differences between the predictions of the model and the real values are the smallest. The covariates with an associated Type 3 p-value lower than 5% have been considered as drivers of the uptakes of biosimilar products (market share in volume of the biosimilars versus the volume of biosimilars plus originator).

## Results

### Incentive policies in place for biosimilars in the selected countries

As of November 2015, the literature search resulted in 680 citations, from which 112 references were considered for inclusion. The full description of the results for the literature review was presented elsewhere [25].

Incentive policies applied to biosimilars are heterogeneous across countries. At the time of the review, pharmaceutical prescription budgets or prescription quotas were reported in place for half of the countries [14,19,39–43] Financial incentives or penalties measures were found in countries applying pharmaceutical prescription budgets and/or prescription quotas, but were reported as not frequently enforced (Belgium, Germany) or enforcement was not documented in the literature (Greece, Italy, Sweden, the UK) [14,39,41,42,44]

Except for Poland, where switching by the physician is generally encouraged [45–47], some countries (Belgium, France, and Italy) do not recommend switching even if they do not prohibit it [14,39], while some other countries might recommend it in some specific cases (e.g., products with same producer (Germany, Sweden) [14], sufficient elapse time between two treatments, or in case of treatment failure (Hungary) [46,47]. In Spain, patients who started biologic therapy usually continue to receive the same medicine, and all decisions should be taken by physicians [13]. In all cases, switching was reported to be the physician’s responsibility. Switching rules were not documented in the literature review for Greece. Some national drug agencies recently positioned in favour of switching under close medical supervision (Paul-Ehrlich-Institut in Germany and some counties in Sweden) [48,49]. In Italy, AIFA stated in its position paper that biosimilars were preferred if they constituted an economic advantage, particularly for treatment-naïve patients [50].

INN prescribing does not apply in the selected countries except in Poland, where the Polish law does not differentiate between generics and biosimilars [11,45]. Automatic substitution of biosimilars by the pharmacists is generally not allowed or recommended. In some countries (Germany, Sweden), substitution is possible for specific groups of biosimilars (e.g., same producer) [14,51–53] In Poland, when no law or guidance applies, automatic substitution may occur [11,54]. France is the first EU country to explicitly authorise by law biosimilar substitution (for naïve patients or to continue a treatment already initiated with the same biosimilar), but implementation decree is still pending and substitution not yet implemented in practice [55]. In all countries, pharmacists are subject to financial incentives or penalties while dispensing drugs, but this is not specific to biosimilars [14,19,38,56]. A patient co-payment system is in place in all countries; for countries where reference price groups exist for off-patent biologics/biosimilars (Germany, Hungary, Poland, Spain) or substitutability criteria apply, i.e., same producer (Sweden), specific co-payment systems are in place to favour use of the cheapest treatment [14,57]. Education and information tools retrieved for biosimilars do not generally target a specific audience (except some specific documentation released to the attention of physicians in Germany [58] and in the UK [19,59,60]), and information is generally scarce on measures in place to enhance biosimilar adoption by the different stakeholders.

Table 6 provides a high-level overview of incentive policies for the selected countries which were used for the regression analysis.

**Table 6.** Overview of key incentive policies in the 10 selected EU countries for biosimilars (Con’t) (as of November 2015).

**Table 6.** 

### Assessment of biosimilar market dynamics

**Figure 1.**
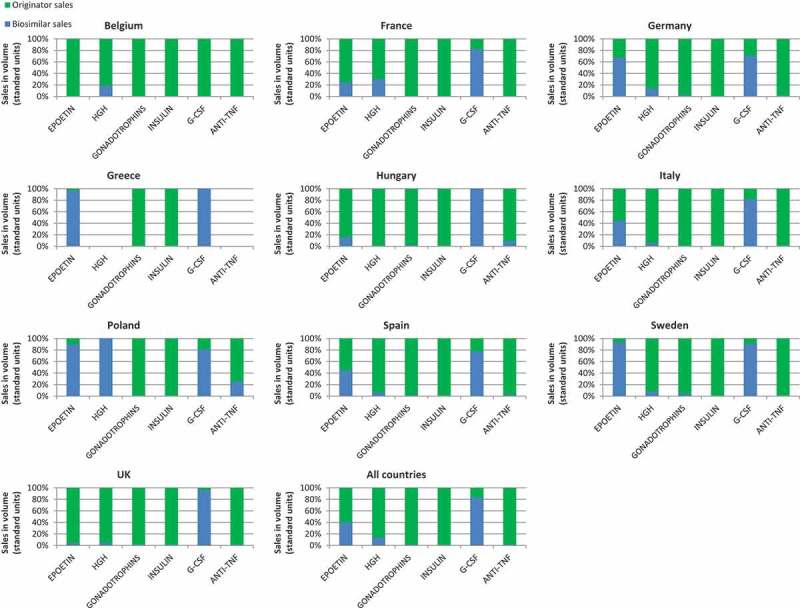
Total 2014–2015 (Q1/Q2) sales in volume of biosimilar and originator products by country and by therapeutic classes.

There is a wide difference in uptake between the different pharmacological classes, the first three old classes on the market (EPO, G-CSF, and HGH) having the highest uptakes (Figure 1).

**Figure 2.**
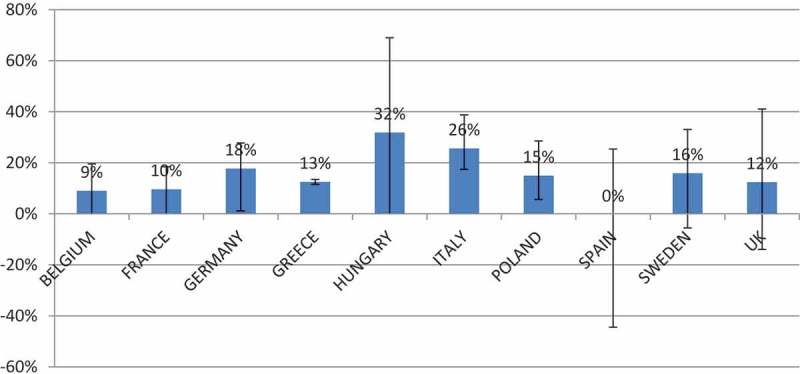
Average biosimilar price discount by country in (%) over originator products.

For these three classes, Poland showed the highest biosimilar penetration compared to other countries, with an uptake level as high as 100% for HGH, 90% for EPO, and 81% for G-CSF. Except for Poland, HGH biosimilar uptake was generally low.

Apart from Poland, Greece and Sweden, followed by Germany, had the highest biosimilar penetrations for EPO (97%, 94%, and 67% respectively). The highest biosimilar uptake were seen in Hungary and Greece for G-CSF (100%), followed by the UK and Sweden (96% and 91% respectively).

The average biosimilar ex-factory price discount was around 15% over originators’, ranging from 0% to 32%, depending on the countries and the products (Figure 2).

### Regression analysis

The model that best fitted the data was a GLM with a normal distribution, an identity link function, with therapeutic class as a random effect. The generic uptakes data used in this model were the set of data from the IGES Institut report. The model performance measures of AIC and RMSE were 49.73 and 0.1852 respectively.

The model results are displayed in Table 7. Results demonstrated that incentive policies and date of first biosimilar market entry were correlated to the biosimilar uptake. The pharmaceutical expenditure per capita and the highest generic uptake were inversely correlated with the biosimilar uptake, i.e., the highest pharmaceutical expenditure per capita and the highest generic uptake being associated with the lowest biosimilar uptake. The average generic price discount over originator and the number of biosimilars showed a trend toward statistical significance for correlation with biosimilar uptake, but did not reach the significance threshold of p < 0.05. The biosimilar price discount over original biologic price, the number of analogues, and the distribution channel were not correlated with the biosimilar uptake, with p value respectively equal to 0.78, 0.50, and 0.33. Therefore, these three variables have no impact on biosimilar uptake.

**Table 7.** Selected generalised linear model results.

Covariates	Estimate sign	P-value
Biosimilar price discounts	–	0.7751 (NS)
Incentive policies	+	0.0283
No. biosimilar products	+	0.0840 (NS)
No. analogue products	–	0.5023 (NS)
Distribution channel	+	0.3310 (NS)
Generic uptake	–	0.0079
Generic price discount	+	0.0607 (NS)
Pharmaceutical expenditure per capita	–	0.0048
First biosimilar market entry date	+	0.0040

Notes: NS: not significant.

Estimate sign describes if the estimates were positive (+) or negative (-).

## Discussion

As reported in the introduction, most of the publications related to the drivers and barriers of biosimilars uptake were rather qualitative research attempting to confront visually and naively the uptake with a sample of attributes in selected countries. Many of these researches were performed at early stages, when few biosimilars were on the market, and for a limited period. Our literature review found one quantitative study using bivariate analysis [18]. To our knowledge, this work is to date the most comprehensive research using a multivariate analysis.

Of note, this is a very dynamic environment where the practice may sometimes differ from the written rules, and our study relies only on secondary research and did not integrate primary research.

This study showed that the number of incentive policies dedicated to enhance biosimilar uptake is an important driver of biosimilar penetration, as already suggested in qualitative studies [16,17,39,40]. The opposite would have been surprising and would have questioned the face validity of the results or the effectiveness of such policies.

The more important the number of biosimilars in a therapeutic class, the higher penetration will be, which is likely related to the largest promotional cumulated effort from more players engaged in the field to support biosimilars; notably, largest penetration of G-CSF biosimilars have been partly attributed to the fact that several biosimilar players (Table 1) have marketed biosimilar of filgrastim, whereas there is only one player marketing the original product [61]. Similarly, a longer time on the market offers a higher opportunity for the biosimilar to be established and therefore to gain market share over the originator, as was the case with the first three biosimilars launched on the market, somatropin, epoetin, and filgrastim showing the largest uptakes.

The pharmaceutical expenditure per capita is inversely correlated to the biosimilar uptake, likely because countries with the highest pharmaceutical expenditure are prone to use more biologics, and are exposed to overall less cost-containment pressure. Moreover, countries like Poland, Greece, or Hungary offer limited access to many original biologics, and when biosimilars are available on the market this enhances use of such products.

Countries which are experiencing high uptake of generics are exposed to less penetration of biosimilars. Such countries may be tempted to address biosimilars like generics and focus primarily on mirroring generic policies. However, such policies have no effect on uptake of biosimilars. Contrary to generics, automatic substitution of biosimilars is generally not allowed or recommended. Moreover, prescribers are usually less price sensitive and are not yet familiar with biosimilars [62]. Indeed, clinicians’ receptiveness and willingness to use biosimilars was reported as an important lever for biosimilar uptake in EU countries [17]. Of note, a weak correlation between biosimilar uptake and price reduction has already been found in one recent IMS study [21], and it has been reported that price discounts was not a key driver of biosimilar uptake [16,20]. It may be that such countries are applying to the biosimilar market the same concepts as to generic products, which are unlikely to work, as the market is totally different, especially with a different competition framework mainly characterised by the need to promote biosimilars and the lack of automatic substitution by the pharmacists.

For each country and for each therapeutic class, biosimilar price discounts were calculated as the differences between the average price per DDD of all biosimilars available on the market (irrespective of dose and pack size) and the average price per DDD of originator products. The averages were computed using the last available ex-factory prices.

The inverse correlation between a high pharmaceutical expenditure and high discount of generic price over the originator on one side versus a low biosimilar uptake on the other side would deserve careful consideration and further research to clarify the underlying rationale. At that stage, we appreciate our interpretation of such findings remains rather speculative, although plausible.

Policy decision-makers may further consider the incentive policies to encourage biosimilar adoption. There could be two reasons why price discount seems to be much less effective. First, at the purchasing level, originators tend in some cases to adjust their prices in order to maintain competitiveness. Second, at the prescribing level, physicians are unlikely to be price driven in the short term when it comes to biosimilars, because the concept is not yet fully established in routine prescription. Moreover, biosimilars will require commercial efforts to be prescribed, and manufacturers may choose to launch biosimilars in countries with higher prices and higher potential for return on investment to get ready to invest in dedicated sales force and medical education.

There is currently a wide gap in terms of policies to support biosimilar adoption between countries. The heterogeneity of healthcare systems, as well as physician prescribing culture and practice, may also be considered when developing such policies. One single large EU set of policies for biosimilars uptake may not be effective for all MS. However, the EU may provide a generic policy framework to enhance biosimilar uptake that should inspire the different MS.

Encouraging the development of biosimilars seems to be a good opportunity, as the number of biosimilars increase the penetration. The number of analogues available does not have an impact on the uptake of biosimilars and at least do not prevent biosimilar uptake.

### Limitations of this research

#### Country selection

As biosimilars are expected to generate substantial healthcare savings, countries were selected based on their level of pharmaceutical expenditure. For budget and technical reasons, we could not access Dutch data, and the Netherlands was replaced by Sweden, a North European country.

Sweden has a well-established framework for pricing and reimbursement of medicines and is frequently used as a reference country when conducting EU cross-country comparisons of drug market access. Besides, through they are different counties, Sweden is an additional example, on top of Italy, Spain, and Germany, to where regionalisation may play a role on biosimilar uptake. Selected countries provide a good representation of the heterogeneity between EU MS in terms of date of entry in the EU [63], gross domestic product per capita [64] (Table 8), as well as health indicators (health status,^1^ ^1^Health status indicators: life expectancy at birth; at 65; mortality from cardiovascular diseases. risk factors,^2^ ^2^Risk factors indicators: smoking in adults; alcohol consumption; obesity in adults; overweight and obesity in children. access to care,^3^ ^3^Access to care indicators: healthcare coverage; share of out of pocket medical expenditure in household consumption; unmet medical care needs; unmet dental care needs; waiting time for cataract surgery; waiting time for knee replacement. quality of care,^4^ ^4^Quality of care indicators: asthma and COPD hospital admission; diabetes hospital admission; case-fatality for acute myocardial infarction; case-fatality for ischemic stroke; cervical cancer survival; breast cancer survival; colorectal cancer survival. health care resources;^5^ ^5^Health care resources indicators: health expenditure per capita; doctors per capita; nurses per capita; hospital beds per capita; MRI units per capita; CT scanners per capita. see the last OECD report *Health at a Glance 2015* [65]).

**Table 8.** Date of entry in the EU and GDP per capita of 10 selected EU countries [53,63].

Country	Date of entry in EU [63]	GDP (US$ per capita), 2014
Belgium	1958	43,723.6
France	1958	39,356.7
Germany	1958	46,393.6
Greece	1981	25,950.4
Hungary	2004	25,060.5
Italy	1958	35,459.2
Poland	2004	24,951.6 (estimated)
Spain	1986	33,637.6
Sweden	1995	45,297.8
United Kingdom	1973	40,209.6

#### Prices

Publicly available prices used in this project may not reflect net prices; indeed, pricing regulations increasingly introduce confidential discounts and tenders in hospitals or at regional levels that may also distort the actual price. However, in practice, in some countries physicians and pharmacists only have access to listed prices and are not able to integrate those discounts in their decisions. At hospital level, the situation may be different, as pharmacists may be aware of the tender price. It is unlikely to be the case for physicians, as tenders or negotiations are confidential procedures. Splitting products according to their channel (hospital, retail, or mixed) allowed to assess if the pricing procedure such as tendering may have impact on biosimilar uptake.

#### Modelling

Unlike the generic market, the low number of biosimilars available and therapeutic classes of biosimilars represents a limitation to modelling the uptake, as it would have been preferred to have a larger number of variables. This was overcome by selecting the model with the best fit, thus strengthening the finding of the results. The best fit selection method was defined *a priori* (based on the AIC and RMSE criteria).

The incentive policies were identified and summed up to generate a score for incentive policies; this assumes that all incentives are equally important, which may not necessarily be true. The alternative method would have been to weight the incentive; such an exercise would have been very subjective and may have created doubt about the validity of such weighted scores. A crude method was then considered as more valid than a weighted one.

#### Countries heterogeneity

Countries appear to be heterogeneous and the reason for uptake of biosimilars in one country may be different in another. Obviously, in Germany, the large number of incentive policies to support biosimilar uptake seems to be the driver, while in countries where the originator was not reimbursed and the biosimilar is reimbursed, biosimilar captures most of the market. This heterogeneity, added to the low number of biosimilars available, represents a potential hurdle for modelling biosimilar uptake, although it did not prevent achieving a good model fit.

Such results should be confirmed in the future, when more biosimilars are available and more time has elapsed from approval to give this market the chance to gain maturity.

## Conclusion

The biosimilar market represents a large opportunity for savings for the society and for a wider use of biologics beyond the restriction of indications mainly driven by prices and affordability of the healthcare systems. Therefore, understanding the drivers of biosimilars uptake becomes a critical issue to inform policy decision-makers.

This is, to our knowledge, the most comprehensive multivariate model developed to identify the drivers of biosimilars uptake. Results are consistent with most previous research, but provide a wider perspective.

Uptakes of biosimilars are very heterogeneous between different therapeutic classes and countries. As expected, incentive policies to enhance uptake remain an important driver of biosimilar penetration. Price discounts of biosimilars over original biologics do not impact the uptake. This should be taken into account by policy decision-makers, who need to get themselves more involved in physician and patient education to ensure better understanding and adoption of biosimilars, and to implement financial incentives for cost-effective prescribing. Simplistic calculation linking the price discount to potential savings is a counter-intuitive strategy, as price discount does not guarantee penetration.

should focus on adopting effective policy incentives to boost the uptake of biosimilars and capture the full potential through savings and wider access to the society. Focusing on biosimilar price discount over originator may not achieve the desirable outcome from an economic and public health perspective.

Future research is warranted when more biologics will go off-patent, more biosimilars are available, and the market gains maturity. This remains a very dynamic market environment, and therefore a close follow-up should be considered to keep policy-makers up-to-date with potential changes in drivers of biosimilar uptake.
